# Determinants of the Mental Health Status of University Students in Bangladesh and Their Coping Strategies: A Cross‐Sectional Study

**DOI:** 10.1002/hsr2.72552

**Published:** 2026-05-25

**Authors:** Md Sanaul Haque Mondal, Rubayat Kabir

**Affiliations:** ^1^ Department of Social Relations East West University Dhaka Bangladesh

**Keywords:** Bangladeshi university students, coping, mental health, psychological distress, student protest, well‐being

## Abstract

**Background and Aims:**

Bangladeshi students participated in the “July Revolution” that ended on August 5, 2024, after a month‐long period. Such protests may negatively impact the mental health of individuals, making effective coping strategies essential in these situations. This study aimed to assess the mental health status and coping strategies of university students after the July Revolution of 2024, and to determine the significant predictors of their mental health status.

**Methods:**

In this cross‐sectional study, data were collected through a web‐based self‐administered questionnaire from 380 university students enrolled in universities across Bangladesh who voluntarily participated. Psychological distress was assessed using the General Health Questionnaire (GHQ‐12) And coping strategies were measured using the Brief‐COPE scale. Bivariable and binary logistic regression models were employed to identify the factors influencing the mental health of university students following the July Revolution. Data analysis was conducted using SPSS Version 26.

**Results:**

The results showed that 86.3% of students experienced poor mental health. The results from the binary logistic regression model demonstrated that the psychological distress among the university students in Bangladesh after the July Revolution was associated with several factors, including the sex of respondents (OR = 2.7, 95% CI = 1.25–5.81; *p* = 0.001), students whose close ones suffered an injury or died (OR = 2.22, 95% CI = 1.06–4.64; *p* = 0.035), those who adopted self‐blame (OR = 1.58, 95% CI 1.24–2.03 *p* < 0.001) and denial coping (OR = 1.39, 95% CI = 1.09–1.76; *p* = 0.007) strategies, and those who did not adopt active (OR = 0.65, 95% CI = 0.49–0.86; *p* = 0.003), and religious coping (OR = 0.70, 95% CI = 0.55–0.89; *p* = 0.004) strategies.

**Conclusion:**

Given the significant psychological impact of the July Revolution on university students in Bangladesh, it is imperative for university administrations to implement targeted mental health interventions. This study recommends providing accessible mental health services by qualified professionals to support students coping with any social movement or other emergencies.

## 1 Introduction

Protests and demonstrations have been increasingly common in recent decades, affecting much of the global population [1]. People are protesting across Africa, Europe, America, and Asia, demanding true democracy, improved public services, employment opportunities, social justice, and an end to abuse, corruption, and austerity, among other things [2].

Concerns over the detrimental effects of social protests on people's mental health have been voiced by many health actors in this highly politicized and emotionally charged environment [3]. Individuals experience health burdens as a result of protests, regardless of whether they have directly participated in such events [4]. Violence associated with social protests has a causal effect on depressive symptoms [3].

Student activism has been historically playing a crucial role in changing social and political platforms in many countries, including Hong Kong [5], Chile [3], and the Philippines [6]. These protests can range from peaceful demonstrations to violent confrontations [7], motivated by various factors, such as political dissatisfaction and social justice demands [8].

The consequences of protest participation affect both social and academic spheres, making students feel more alone and distressed [9], particularly in environments where institutional support is lacking [10]. Prior research has indicated elevated levels of anxiety, depression, and post‐traumatic stress disorder (PTSD) among students due to their exposure to protests in different countries [3, 11, 12].

Students' protests have raised concerns about the mental health costs for affected students [9]. There is limited research examining the relationship between student protests and mental health status [3, 4, 5, 13]. The majority of published research has been carried out in high‐income countries [11], leaving a notable gap in research from low‐ and middle‐income countries regarding the association between mental health and social protests. Although social unrest and protests are worldwide issues [14], there is increasing concern in low‐ and middle‐income nations [3].

Bangladesh, a developing country, has a longstanding tradition of student protests, commonly known as *Chatro Andolon* in Bengali. The role of student protests was found to be remarkable in the 1952 Language Movement, the 1971 Liberation War, and the return of democracy in 1990. Over the past 10 years, the country has witnessed multiple waves of student protests. In 2018, there were two student‐led movements in the country: a movement for improved road safety and quota reform in the civil services [15, 16].

In 2024, Bangladesh faced significant challenges due to the quota reform movement in the civil services. The movement began with urban students in Dhaka and spread nationwide, leading to the establishment of student committees throughout the country [17]. This movement garnered support from a diverse group of students. After almost a month of nationwide student mass protests, the movement concluded on August 5, 2024, with the previous government's ousting [18, 19]. During this monthlong protest, people in Bangladesh, particularly students, were exposed to physical and mental abuse either in person or on social media platforms. Participation in protests and collective actions can have a negative psychological effect on the protesters [1]. Therefore, it is imperative to address potential mental health issues in Bangladesh, especially for student protestors and the families of students killed or injured, as they may suffer from PTSD, anxiety, depression, and prolonged grief disorder.

Earlier studies in Bangladesh have assessed the mental health of university students [20, 21, 22]. Although the current literature on the July Revolution has concentrated on the mental health of students [23], and the adult population [24, 25, 26], there has been relatively scant focus on identifying the factors associated with psychological distress among university students following the nationwide student protests. The July Revolution can have a detrimental effect on the mental health of university students. Therefore, a rigorous analysis of the determinants of mental health among university students after the protest using representative data is required.

Against this background, this study examined [1] the overall mental health status of university students in Bangladesh following the July Revolution [2], the coping strategies employed by university students, and [3] the ways in which socio‐economic status, protest exposure, and various coping strategies aid students in managing their psychological distress. It is vital to obtain a deeper understanding of how the July Revolution affected university students' mental health in this unique context of Bangladesh.

### 1.1 The 2024 July Revolution in Bangladesh

Looking back, in 1972, shortly after Bangladesh received independence, the quota system allocated 80% of government positions to individuals affected by the war. However, several amendments have been made since then. Before 2018, the quota system reserved specific percentages in higher education seats and civil service jobs for various groups, including individuals with physical disabilities (1%), minorities (5%), people from backward districts (10%), women (10%), and freedom fighters (30%), resulting in a cumulative reservation of 56% [27]. Eventually in 2018, significant protests erupted against perceived injustices related to job shortages caused by the quota system, particularly the 30% quota allocated for the grandchildren of the independence fighters of 1971 [28]. Nevertheless, the protest ended when the government declared the cancellation of the quota system.

The 2024 Quota Movement in Bangladesh (herein abbreviated as the July Revolution) is one of the largest demonstrations in recent decades. It commenced after the High Court's declaration on June 5, 2024, regarding the reinstatement of quotas for the descendants of freedom fighters from the 1971 liberation war in Bangladesh. Students, particularly those studying in universities, strongly objected to this decision. In reaction to the High Court's ruling, students gathered at the Central *Shaheed Minar* (national monument) in Dhaka, the nation's capital. Peaceful protests began with students at public universities and quickly spread across the nation due to their extreme intensity. Subsequently, students from private universities joined the protest.

The provocative comments and harsh government measures that followed the protest fueled the protestors' anger even more. At least six people lost their lives in the violent altercations between protesting students and law enforcement on July 16 [29]. To suppress the protest, the government closed all educational institutions. The authorities' use of arbitrary, illegal arrests and detentions, internet shutdowns, brutal violence, and other human rights violations caused the situation to quickly spiral out of control and turn into a large‐scale protest. This led to numerous deaths and injuries, a nationwide shutdown, and clashes between protesters and law enforcement. The government imposed curfews and deployed the military forces [28].

The Supreme Court ruled on an appeal on July 21, 2024 cutting the war veterans' quota to 5%, allocating 2% to members of ethnic minorities, transgender individuals, and disabled people, and 93% of jobs based on merit. Despite the government's acceptance of the Supreme Court's decision, demonstrations continued, advocating for further political reforms and accountability for the violence. The death toll escalated to 148 in a mere 5 days (July 16–20, 2024), and a total of 550 people were arrested across the country [29].

On 5 August 2024, thousands attempted to gather at several intersections in Dhaka and enter the capital, in defiance of the curfew. Violent clashes erupted with ruling party supporters and law enforcement. In the end, the Prime Minister agreed to resign and fled the country on a helicopter. To celebrate the Prime Minister's downfall, thousands of people congregated in the streets [29]. According to the Office of the High Commissioner for Human Rights report from July 15 to August 5, at least 1,400 people died and over 11,700 were injured [30].

### 1.2 Factors Associated With Psychological Distress

Researchers have extensively used psychological distress as a mental health indicator [31, 32]. Anxiety, depression, suicidal ideation, and negative reaction to stressful events caused by everyday life are signs and symptoms of psychological distress [33]. Protests have the potential to adversely affect an individual's mental health, so effective coping is necessary to prevent or reduce psychological distress and to deal with crises [31, 34, 35].

Previous research that revealed poor mental health among university students has included factors such as sex, age, type of university, academic year, income sources, exposure to violence, and support from close ones [13, 33, 36, 37, 38, 39, 40, 41, 42, 43, 44, 45, 46, 47]. The unpredictability of social unrest has caused severe distress such as depression and anxiety among female university students [34, 38]. Psychological distress is prevalent among the younger age groups of students [45, 48]. Financial independence is linked to reduced psychological distress [31, 45, 48, 49] and student's with inadequate personal income is more susceptible to anxiety and depression [38, 41].

Additionally, mental health disorders were associated with the students' academic year at university [31]. Furthermore, students attending public universities were found to be more depressed than students of private universities [43, 48]. Moreover, social media use has also been identified as a risk factors for poor mental health [42]. In the light of this information, the first hypothesis was proposed:

Hypothesis 1 The psychological distress among university students varies according to their socio‐demographic characteristics.

Other risk factors associated with poor mental health include exposure to violence [42], the intensity and duration of protests [11], the presence of support systems [3], and individual coping strategies [10]. Protesters exposed to injurious events had significantly higher levels of PTSD and depression than non‐exposed individuals [12]. However, Pancer et al. [50] found an insignificant association between protest participation and depressive symptoms. Therefore, the second hypothesis was proposed:

Hypothesis 2 Students' exposure to protest is positively associated with psychological distress.

Besides, coping is a humans' behavioral and cognitive responses to internal and external demands under a stressful circumstance [51, 52, 53, 54]. Most coping frameworks converge on the two core dimensions identified by Folkman and Lazarus: emotion‐focused and problem‐focused coping [52, 55]. Emotion‐focused coping is reactive, aiming to regulate emotional responses to stress, whereas problem‐focused coping is proactive, involving direct efforts to address the source of distress [51, 52]. A third dimension, avoidant coping, has also been proposed, marked by disengagement through distraction, social diversion, or substitute behaviors. Of these, problem‐focused coping is generally considered the most adaptive [56]. Some cope by avoiding situations or people who cause them distress or resolving the dilemma, while others find solace in spiritual practices or life changes [35, 57]. On the flip side, protest involvement can lead to the development of strong social networks and a sense of solidarity, which can increase the capacity to cope with adversity (Drury et al., 2009). Based on the literature, the following can be inferred:

Hypothesis 3 Psychological distress is negatively associated with social support and coping strategies.

## 2 Methods and Materials

### 2.1 Study Design and Population

We adopted a cross‐sectional study design to collect data from university students in Bangladesh from August 20 to September 14, 2024, which is fifteen days after the end of the students' protest.The timing allowed us to capture relatively immediate psychological impacts.

We collected data using snowball sampling, a non‐probability sampling technique. This method was chosen because it was difficult to access a comprehensive sampling frame during the nationwide protest, and there was an urgent need to capture timely data. A self‐administered web‐based questionnaire using Google Forms was shared with students through social media platforms, particularly via Facebook Messenger and WhatsApp. Respondents were requested to share the survey link with other university students, which helped reach a broader and a more diverse group despite the non‐random sampling method. The language of the questionnaire was in English as it is used as the medium of instruction in Bangladeshi universities and is commonly understood by students across both public and private universities.

According to the University Grants Commission in Bangladesh, the total number of students in 2021 was 44,41,717. With a 5% margin of error and a 95% confidence interval, the required number of samples for our study was 385. Three hundred eighty‐five students from 48 institutions (22 government and 26 private) responded to our survey questionnaire. However, we removed five records due to ineligibility, because one respondent lived outside Bangladesh, and four respondents had already graduated and were not current students. Finally, we included 380 student records for analysis. A pilot study was conducted with seven university students to determine the reliability of the instruments. After a series of discussions with them, we finalized the questionnaire to make it more user‐friendly. These seven responses were excluded from the analysis.

### 2.2 Inclusion and Exclusion Criteria

The inclusion criteria for the survey were university students in Bangladesh who were currently enrolled in either private or public universities, had firsthand experience with the 2024 student protest, had access to the internet, and were able to complete a web‐based questionnaire in English. Those who were outside Bangladesh or had graduated were ineligible to participate in this study.

### 2.3 Instruments

We designed the questionnaire to assess university students' exposure to protests, their perceived support from family and the university, their mental health status, and their coping strategies. In addition, we asked the respondents one open‐ended question: “Would you like to share anything related to the quota movement?”, allowing them to express their thoughts freely.

#### 2.3.1 Dependent Variable

Symptoms of mental health status were measured using Goldberg's General Health Questionnaire‐12 (GHQ‐12), a self‐administered questionnaire designed to detect mental illness [58]. The GHQ‐12 has been widely used to assess the mental health status of adults in many countries [39, 59, 60]. Studies have proven that the GHQ‐12 is valid and reliable in the context of Bangladesh [43, 61].

The Shapiro‐Wilk test was used to test normality in the data set. If the *P*‐value is below 0.05, the data deviate from a normal distribution; if the *P*‐value exceeds 0.05, it suggests that the data are normally distributed [62]. We assessed the normality of the GHQ‐12, and the results rejected the null hypothesis of normality. This result is usual because the GHQ‐12 scoring system is discrete in nature. The reliability of the GHQ‐12 items were tested by Cronbach's alpha. The Cronbach's alpha value of the GHQ‐12 in our study was 0.751, indicating the instrument was reliable.

We used the bimodal GHQ‐12 scoring method (0‐0‐1‐1), which was summed to yield an overall score range of 0–12. The GHQ's cut‐off of > 2 used to indicate possible mental health conditions [43, 63]. The mental health status of the students was the dependent variable of this study, which was grouped into two levels: psychological distress (sum of GHQ‐12: > 2) and not having psychological distress (sum of GHQ‐12: ≤ 2).

#### 2.3.2 Explanatory Variables

A multitude of factors influence the mental health of university students, and there is no agreed framework for choosing explanatory variables. Based on literature review and discussion with the students, socio‐demographic characteristics, exposure to protests, perceived social support, and coping strategies were considered the explanatory variables for this study.

##### 2.3.2.1 Socio‐Demographic Characteristics

The model included the respondents' sex, personal income, university type, study level, and affiliation with any university clubs.

##### 2.3.2.2 Exposure to Protest

To capture the exposure to protest, this study considered three variables [1]: physically present in the protest [2], personal injury, and [3] injuries/death of close ones during the protest. Each of these three factors had a dichotomous response option.

##### 2.3.2.3 Perceived Social Support

Respondents' perceived level of support from their family members and university during and after the protest, which comprised of four factors [1]: level of support from family members during the protest [2], level of support from family members after the protest [3], level of support from the university during the protest, and [4] level of support from the university after the protest. The response alternatives for the perceived support variables included never = 0, sometimes = 1, and always = 2. We developed a perceived social support variable by summing these four indicators, which produce a possible total score of 0–16. Cronbach's alpha value for social support was 0.60, indicating a moderate level of reliability, and the index for the social support variable was not normally distributed.

##### 2.3.2.4 Brief‐COPE

The Brief Cope inventory was used for this study to assess the coping strategies [64]. The Brief Cope measures include 28 items addressing the 14 coping strategies [1]: active coping [2], planning [3] use of emotional support [4] use of informational support [5], positive reframing [6] acceptance [7] religion [8] humor [9] venting [10] denial [11], substance use [12] behavioral disengagement [9] self‐distraction [13] self‐blame. Each item asks respondents to indicate whether they generally engage in a particular coping activity with four response options: ‘I don't usually do this at all’ (=1), “I usually do this a little bit” (=2), “I usually do this a medium amount” (=3), or “I usually do this a lot” (=4) [64]. The sum of each coping strategy should be 0‐8. The reliability of the Brief‐COPE was tested by Cronbach's alpha. The Cronbach's alpha value of Brief‐COPE measure is 0.762, indicating the instrument was reliable. The normality of the 14 coping strategies was assessed using the Shapiro‐Wilk test, and none of these measures were found to be normally distributed.

### 2.4 Data Analysis

The data analysis was performed using Statistical Product and Service Solutions (SPSS) version 26.0. Descriptive statistical analyses (mean, standard deviation [SD], frequency) were performed to comprehend the characteristics of the respondents, the prevalence of psychological distress, and the coping strategies. The dependent variable of this study was whether a respondent had symptoms of psychological distress or not. To determine the dummy variable, respondents with psychological distress were assigned 1, and those without were assigned 0. If the sum of GHQ‐12 was > 2, it was an indication of psychological distress. Since the dependent variable is binary, multivariate binary logistic regression was used [65] to model the influence of explanatory variables (socio‐demographic factors, exposure to violence, and coping strategies) on mental health status of university students. The binary logistic regression model reports the odds ratios (OR) and associated 95% confidence intervals (CI) for each explanatory variable. Model fitness was checked using the Hosmer‐Lemeshow test, which yielded a *p*‐value of 0.552, suggesting that the model was fitted to our data. All statistical analyses were conducted employing a two‐sided test, and statistical significance was set as a *p*‐value of less than 0.05.

### 2.5 Ethical Considerations

The study obtained ethical clearance from the Ethical Review Committee, East West University. The decision to participate in this study was completely voluntary. The respondents were asked to provide their informed consent electronically before proceeding with the main items of the survey. Respondents were free to withdraw at any time during the survey. The respondents' email addresses and contact numbers were not collected in order to maintain their anonymity and confidentiality. This study followed the ethical guidelines of the Helsinki Declaration.

## 3 Results

### 3.1 Respondents' Characteristics

Table 1 shows the socio‐demographic characteristics of 380 respondents from 48 universities in Bangladesh. The ages of the respondents ranged from 18 to 30 years (mean=22.58, SD = 1.96). Around 56.6% of respondents were female. Most respondents (94.7%) were Muslims and were unmarried (91.9%). Around 87.1% of the respondents were undergraduate students. In terms of occupation, 34.2% of respondents had income from full/part‐time jobs, businesses, or tuition. This means that the rest of them (65.8%) solely depended on their parents as the primary source of income. Over four‐fifths of the respondents (82.4%) lived with their families during the protest. Most respondents (80.0%) were from private universities. Half of the respondents (50.5%) reported participating in club activities within their university.

**Table 1:** Socio‐demographic characteristics of respondents (*N* = 380).

Demographics	Frequency (%)
Age (years)	
18–22 years	205 (53.9%)
23–30 years	175 (46.1%)
Sex	
Male	165 (43.4%)
Female	215 (56.6%)
Type of university	
Private	304 (80%)
Public	76 (20%)
Marital status	
Single	349 (91.8%)
Married	31 (8.2%)
Academic level	
Undergraduate	331 (87.1%)
Postgraduate	49 (12.9%)
Religion	
Islam	360 (94.7%)
Hindu	18 (4.7%)
Christian	2 (0.5%)
Have a personal income	
Yes	130 (34.2%)
No	250 (65.8%)
Engaged in club activities	
Yes	192 (50.5%)
No	188 (49.5%)

*Source:* Survey, 2024.

Among the respondents, 73.9% had physically participated in the protest, and 96.1% were active on social media through posting and sharing protest‐related information. During the protest, a small number of respondents (10%) sustained injuries. Over half of the respondents (55.3%) reported that their close ones were injured or died during the protest.

**Figure 1:**
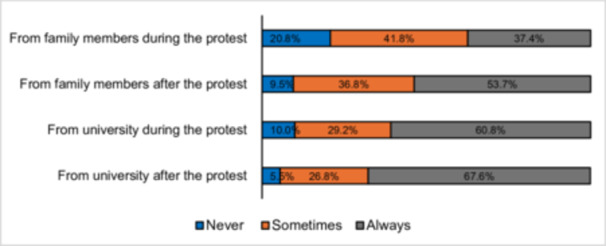
Support from family members and university during the protest (*N* = 380). *Source:* Survey, 2024.

Figure 1 presents the level of support university students received from their family members and their universities during and after the July Revolution. Respondents informed that the support received from family members was substantial but somewhat lower during the protest (79.2%) compared to after the protest. Similarly, support from universities was relatively high during the protest (90%) and increased further after the protest ended (94.4%).

### 3.2 Prevalence of Mental Health Outcomes

Table 2 lists the item‐wise distribution of the GHQ‐12 scores. Nearly two‐thirds of respondents who participated in the survey mentioned having sleep‐related issues (item 2). Almost half of the respondents (48.8%) were unable to overcome their difficulties (item 6); (*n* = 193) were losing confidence in themselves (item 10); and (48%) thought of themselves as worthless (item 11). More than half of the respondents (56.7%) reported feeling constantly under strain (item 5) and (60%) were unhappy and depressed (item 9).

**Table 2:** Item‐wise descriptive statistics of GHQ‐12 (*N* = 380).

Items of GHQ‐12	Mean ± SD
Item 1: Been able to concentrate on what you are doing	0.55 ± 0.49
Item 2: Lost much sleep over worry	0.66 ± 0.48
Item 3: Felt you were playing a useful part in things	0.55 ± 0.49
Item 4: Felt capable of making decisions about things	0.46 ± 0.49
Item 5: Felt constantly under strain	0.57 ± 0.49
Item 6: Felt you could not overcome your difficulties	0.49 ± 0.50
Item 7: Been able to enjoy your normal day‐to‐day activities	0.53 ± 0.49
Item 8: Been able to face up to your problems	0.44 ± 0.49
Item 9: Been feeling unhappy and depressed	0.60 ± 0.49
Item 10: Been losing confidence in yourself?	0.51 ± 0.50
Item 11: Been thinking of yourself as a worthless person	0.48 ± 0.50
Item 12: Been feeling reasonably happy, all things considered	0.54 ± 0.49

*Source:* Survey, 2024.

**Figure 2:**
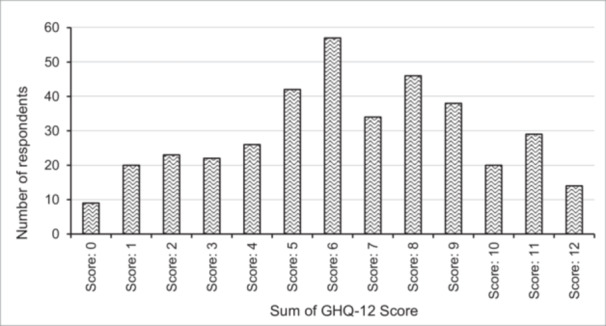
Frequency distribution of the sum of GHQ‐12 scores (*N* = 380). (*Source:* Survey, 2024).

Figure 2 shows the total GHQ‐12 scores (sum of the 12 items) and the prevalence of psychological distress among university students. A score of 6 indicated the highest prevalence among 15% of the students, followed by 12.1% (score: 8) and 11% (score: 5). The mean value of psychological distress was 6.37 (SD 3.08), and 86.4% of respondents had a total score above 2, indicating a high risk of psychological distress among the university students after the protest.

### 3.3 Coping Strategies

**Figure 3:**
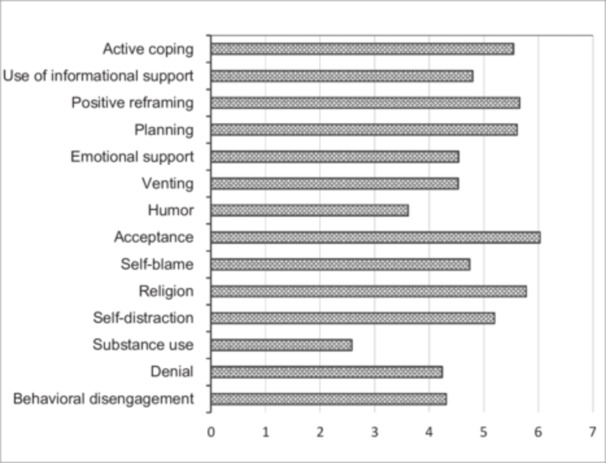
Coping strategies according to Brief‐COPE scale (*N* = 380). *Source:* Survey, 2024.

The self‐reported coping strategies are presented in Figure 3. To manage psychological distress following the protest, the most frequently reported coping strategies were active coping (5.54), positive reframing (5.66), planning (5.61), acceptance (6.03), and religion (5.78). The least reported coping strategies were substance use (2.58) and humor (3.62), respectively. For five of the 14 coping strategies, the reported use varied significantly between female and male respondents.

### 3.4 Determinants of Mental Health Status Using Multivariate Logistic Regression

To identify the factors associated with psychological distress among university students following the protest, we used a binary logistic regression model. The model was used to examine the following variables: socio‐demographic characteristics (sex, personal income, type of university, level of study, and involvement in club activities), exposure to protest (direct exposure to protest, personal injury, and respondents close one injured/died), support from family members and university (sum of support measures), and coping strategies (Brief COPE measures) (Table 3).

**Table 3:** Summary statistics of selected variables for binary logistic regression (*N* = 380).

Variable	Variable descriptions	Mean ± SD
Socio‐demographic		
Sex	Sex of respondent: male = 0, female = 1	0.57 ± 0.50
Income	Have personal income: yes = 1, no = 0	0.34 ± 0.48
University type	Type of university: private = 1, public = 0	0.80 ± 0.40
Study level	Level of study: undergraduate = 1, postgraduate = 0	0.87 ± 0.34
Club engagement	Engaged in any club activities within the university system: yes = 1, no = 0	0.51 ± 0.50
Exposure to protest		
Field involvement	Physically present in the protest: yes = 1, no = 0	0.74 ± 0.44
Self‐injury	Personal injury during the protest: yes = 1, no = 0	0.10 ± 0.30
Close one injured/died	Close one injured/died during the protest: yes = 1, no = 0	0.55 ± 0.50
Perceived support		
Perceived support	Respondents perceived support received from family members and the university during and after the protest (continuous).	5.74 ± 1.80
Coping strategies		
Active coping	Active coping (continuous)	5.54 ± 1.558
Use of informational support	Use of informational support (continuous)	4.79 ± 1.875
Positive reframing	Positive reframing (continuous)	5.66 ± 1.661
Planning	Planning (continuous)	5.61 ± 1.621
Emotional support	Emotional support (continuous)	4.54 ± 1.712
Venting	Venting (continuous)	4.53 ± 1.609
Humor	Humor (continuous)	3.62 ±
Acceptance	Acceptance (continuous)	6.03 ± 1.637
Self‐blame	Self‐blame (continuous)	4.74 ± 1.988
Religion	Religion (continuous)	5.78 ± 1.900
Self‐distraction	Self‐distraction (continuous)	5.19 ± 1.640
Substance use	Substance use (continuous)	2.58 ± 1.342
Denial	Denial (continuous)	4.24 ± 1.780
Behavioral disengagement	Behavioral disengagement (continuous)	4.31 ± 1.674
Mental health status		
Mental health	Mental health status of respondents using GHQ‐12	0.86 ± 0.344

*Source:* Survey, 2024.

The binary logistic regression model is shown in Table 4. The model was statistically significant. The Hosmer–Lemeshow chi‐square test did not yield a statistically significant result for the model, suggesting that the model is fit for prediction. The model explained 33.9% (Nagelkerke R²) of the variance in the mental health status of university students.

**Table 4:** Determinants of mental health status of university students (1 = psychological distress, 0 = not having psychological distress).

Variables	*β*	*p* value	OR	95% CI	
				Lower	Upper
Sex					
Male		Reference			
Female	0.99	0.011	2.70	1.25	5.81
Have income					
No		Reference			
Yes	0.08	0.831	1.09	0.50	2.37
University type					
Public		Reference			
Private	0.34	0.448	1.41	0.58	3.42
Study level					
Postgraduate		Reference			
Graduate	−1.24	0.140	0.29	0.06	1.50
Club engagement					
No		Reference			
Yes	−0.20	0.575	0.82	0.40	1.66
Field involvement					
No		Reference			
Yes	0.27	0.541	1.30	0.56	3.07
Self‐injured		Reference			
No					
Yes	0.65	0.299	1.92	0.56	6.56
Close one injured/died					
No		Reference			
Yes	0.80	0.035	2.22	1.06	4.64
Perceived support	−0.02	0.836	0.98	0.79	1.21
Active coping	−0.43	0.003	0.65	0.49	0.86
Use of informational support	0.08	0.551	1.08	0.84	1.39
Positive reframing	−0.05	0.732	0.96	0.73	1.24
Planning	0.001	0.993	1.00	0.76	1.32
Emotional support	−0.17	0.196	0.84	0.65	1.09
Venting	−0.10	0.401	0.90	0.71	1.15
Humor	−0.20	0.086	0.82	0.65	1.03
Acceptance	0.08	0.581	1.08	0.82	1.43
Self‐blame	0.46	< 0.001	1.58	1.24	2.03
Religion	−0.35	0.004	0.70	0.55	0.89
Self‐distraction	0.18	0.113	1.20	0.96	1.50
Substance use	0.27	0.252	1.30	0.83	2.05
Denial	0.33	0.007	1.39	1.09	1.76
Behavioral disengagement	−0.03	0.767	0.97	0.77	1.21
Constant	3.23	0.040	25.34		

The findings show that female respondents were more likely to exhibit poor mental health (OR = 2.7, 95% CI = 1.25–5.81; *p* = 0.001) (Table 4). This study found an insignificant association between the personal income of respondents (OR = 1.09, 95% CI = 0.50–2.37; *p* = 0.831), type of university (OR = 1.41, 95% CI = 0.58–3.42; *p* = 0.448), and level of study (OR = 0.29, 95% CI = 0.06–1.50; *p* = 0.140) and psychological distress. Similarly, involvement in club activities within the university did not significantly influence psychological distress (OR = 0.82, 95% CI = 0.40–1.66; *p* = 0.575), however, the relation was found to be negative.

Among the exposure to protest variables, although not significant, physically attending the protest (OR = 1.30, 95% CI = 0.56–3.07; *p* = 0.541) and personal injury (OR = 1.92, 95% CI = 0.56–6.56; *p* = 0.299) during the protest were positively associated with mental distress. A significant positive association was observed between psychological distress and the injury or death of a close friend or relative during the protest (OR = 2.22, 95% CI = 1.06–4.64; *p* = 0.035). Additionally, perceived support from family members and the university was negatively associated with mental distress, though not statistically significant (OR = 0.98, 95% CI = 0.79–1.21; *p* = 0.836).

In terms of coping strategies, psychological distress was negatively associated with active coping (OR = 0.65, 95% CI = 0.49‐0.86; *p* = 0.003) and religious coping (OR = 0.70, 95% CI = 0.55–0.89; *p* = 0.004). Likewise, positive reframing (OR = 0.96, 95% CI = 0.73–1.24; *p* = 0.732), emotional support (OR = 0.84, 95% CI = 0.65–1.09; *p* = 0.196), venting (OR = 0.90, 95% CI = 0.71–1.15; *p* = 0.401), humor (OR = 0.82, 95% CI = 0.65–1.03; *p* = 0.086), and behavior disengagement (OR = 0.97, 95% CI = 0.77–1.21; *p* = 0.767) showed a negative relationship with psychological distress, although not statistically significant.

Our findings revealed a significant positive association between self‐blame (OR = 1.58, 95% CI 1.24–2.03; *p* < 0.001) and denial (OR = 1.39, 95% CI = 1.09–1.76; *p* = 0.007), indicating that individuals who score higher on these coping measures are more likely to experience psychological distress. The use of informational support (OR = 1.08, 95% CI = 0.84–1.39; *p* = 0.551), planning (OR = 1.00, 95% CI = 0.76–1.32; *p* = 0.993), acceptance (OR = 1.08, 95% CI = 0.82–1.43; *p* = 0.581), self‐distraction (OR = 1.20, 95% CI = 0.96–1.50; *p* = 0.113), and substances used (OR = 1.30, 95% CI = 0.83–2.05; *p* = 0.252) were positively associated but did not significantly correlate with psychological distress.

## 4 Discussion

Following the nationwide student protest in Bangladesh, the present study examined university students' mental health and coping strategies using the GHQ‐12 and Brief‐COPE questionnaires, respectively, to determine the factors influencing mental health status. The study found that 86.3% of the respondents experienced psychological distress. This prevalence of psychological distress was higher than earlier studies conducted in Bangladesh [66, 67], but comparable to Chowdhury et al. [68]. These findings are crucial as the students' movement has affected the whole country, and students are at risk of developing mental disorders.

### 4.1 Socio‐Demographic Characteristics

Our findings indicate that the mental health condition of female students were worse than that of male students, which is consistent with previous studies [23, 34, 38, 67, 69, 70, 71]. This may be attributed to female students being more susceptible to mental health difficulties than their male counterparts [72, 73] and exhibiting overall greater mental vulnerability [13, 74]. Nonetheless, our findings contradict a study conducted in Egypt, which indicated that male students exhibited higher levels of depression than their female counterparts [36].

Mental health problems among university students may also be associated with a lack of financial support from their family [75]. However, this study did not find any relationship between personal income and mental health status. These findings align with Mondal [23], who did not find any association between psychological distress and personal income among the students in Bangladesh after the July Revolution. However, the findings are inconsistent with previous research conducted in Bangladesh [48] and other countries which reported that students with personal income had better mental health than those who were economically dependent on their families [31, 38, 49, 75]. This could be because, regardless of the students' socio‐economic background, a large majority of the respondents were involved in the July Revolution. Although prior studies indicate that students at public universities experience poorer mental health than private university students [43], our study did not find a significant association between mental health and type of university. It is possible to infer that private university students may have poorer mental health than public university students, as the coefficient was positive, although not statistically significant. Typically, political activities are restricted in private universities in Bangladesh [76]. Moreover, private universities generally have limited concerns regarding campus violence, political affiliations, delays in graduation, dormitory arrangements, and so on. However, during the July Revolution, students from private universities played a pivotal role in the protest, and their participation greatly strengthened the movement when students at public universities were asked to vacate their university dormitories.

Similarly, the level of study (undergraduate vs. postgraduate) did not significantly influence the respondents' mental health conditions. A previous study in Bangladesh found no significant association between the academic year and mental health disorders [43]. However, as the coefficient was negative, it may be inferred that postgraduate students had poorer mental health, possible because they are prospective jobseekers who are worried about their future [31].

Student clubs may contribute to a more positive campus environment [77]. Although our study did not observe a significant association between engagement in club activities and respondents' mental health status, the negative association suggests that club engagement may help reduce stress through peer support, educational opportunities, and social interaction on campus.

### 4.2 Exposure to Protest

The nature of protest engagement and the level of exposure to violence during protests are significant risk factors for mental health outcomes [26, 78, 79]. Our findings regarding physical involvement in the protest and personal injury are inconsistent with previous studies [1], as we were unable to find any significant associations between these factors and psychological distress. This indicates that the incidence of probable depression increases irrespective of one's personal participation in the protests, suggesting potential spillover effects on the community [42]. This suggests that individuals who did not take part in the collective action can still experience mental health effects. In the context of Bangladesh, this was probably because, although many respondents did not participate in the protests due to family or other obligations, they remained highly active on social media. One of the respondents who did not physically participate in the protest stated that:

I remember, I couldn't sleep at night from 16 July to 6 August. Internet was not available. I couldn't talk or share anything with my family members and friends. I am facing mental trauma by seeing some dead bodies in the roads. Sometimes I just cry for no reason (female, age 24, undergraduate student).

However, a significant positive association was found between a close one injured or died during the protest and psychological distress. This finding corroborates earlier studies [12]. One of the respondents who was physically present in the protest and whose friend got injured stated that:

When I went to protest in front of my university, the police opened fire on people. I saw one of my university brothers was shot. Injured people are running towards the hospitals. When the internet was cut off throughout the country, I could not sleep at night because of the fear of what was happening and how many brothers were dying (female, age 21, undergraduate student).

### 4.3 Perceived Support

The findings from our study, although not statistically significant, show that support from family members and university could reduce psychological distress. In fact, social support is considered as a significant protective factor in reducing psychological distress [41, 80, 81]. This could be better explained by the social support deterioration model which suggests that the reducing social support, especially after a protest can lead to both immediate and delayed psychopathology, particularly in human‐induced events such as collective actions [42].

### 4.4 Coping Strategies

To counteract the effect of the nationwide student protest, this study also investigated which coping strategies were related to poor mental health status. The findings suggest that active coping was negatively related to psychological distress, indicating that students who adopted this coping strategy showed good mental health. These findings are consistent with previous literature, which reported that active problem‐focused coping is associated with lower distress [24]. However, positive reframing, use of informational support, and planning were not associated with the mental health of Bangladeshi university students after the protest. Our findings align with previous studies [82] that reported positive reframing was not associated with the mental health of Ukrainians after the Russia‐Ukraine war. Among these coping strategies, positive reframing was negatively associated. The reasons why positive reframing coping strategies were not adopted by the university students may be because the students' protest escalated so quickly that the students were unable to see it positively [82].

Coping through self‐blame was positively correlated with psychological distress, which is consistent with previous study [83]. The significant negative association between religious coping and psychological distress indicated that respondents who had adopted religious coping showed good mental health. Our findings are inconsistent with a previous study conducted in Palestine [84], which reported that using religion as coping increased the probability of having depressive symptoms. In our study, this is particularly relevant, as over 94.7% of respondents were Muslims. Previous research suggests that Muslim students use religiosity to cope with psychological distress through prayer, belief and faith, patience, and expressing thankfulness to the Almighty [85]. One of the respondents who showed good mental health stated that:

I was praying a lot so that things would get better (female, age 23, undergraduate student).

Denial coping mechanisms involve ignoring the reality of a situation to avoid psychological distress. Our study found a positive significant association between denial coping and psychological distress. This is due to the fact that denial prevents integration and delays support and coping, which may increase psychological distress [86].

### 4.5 Limitations of This Study

This present study has some limitations. This study utilized a cross‐sectional design that constrains the ability to infer causality. The self‐reported questionnaire may introduce response bias. The findings of our study are limited to data collected immediately after the July Revolution, a period when most universities, especially public universities, were closed. Students without access to mobile devices were excluded, potentially introducing selection bias. The use of snowball sampling may have led to sampling bias. Students from religious institutions (*Fazil and Kamil Madrasha*) did not participate in this study, potentially missing their health status. Nevertheless, this study provided evidence about the impact of the protest on the university students' mental health. Future studies should examine the mental health impact of the protest on the secondary and higher secondary students who were also active participants in the protest.

## 5 Conclusion

The study offers empirical evidence on the mental health symptoms of university students soon after the student protests in Bangladesh in July 2024. The prevalence of psychological distress among university students after the protest is high, with over four‐fifths showing poor mental health. Female students exhibited poorer mental health conditions than males.

Students who were injured or lost loved ones during the protest showed worse mental health conditions. University students adopted various coping strategies, such as religion, active coping; self‐blaming, and denial to cope with the psychological distress soon after the protest.

To address these challenges, it is strongly recommended that university administrations across the country take necessary steps to provide mental health services through mental health specialists to improve students' well‐being. Furthermore, female students, among whom mental health problems are more prevalent, should receive special attention. Our data also indicates the need to familiarize university students with effective coping strategies through workshops and seminars aimed at destigmatizing mental health issues and encouraging students to seek assistance without hesitation. Engaging students in various extracurricular activities is also essential. Finally, a strong monitoring and evaluation system should be developed to understand how mental health programs support students in coping with stress and build resilience.

## Author Contributions

**Md Sanaul Haque Mondal:** conceptualization, methodology, software, data curation, supervision, formal analysis, validation, visualization, project administration, writing – review and editing; writing – original draft, investigation. **Rubayat Kabir:** conceptualization, methodology, writing – original draft, writing – review and editing, investigation.

## Funding

The authors have nothing to report.

## Ethics Statement

The Helsinki Declaration was followed in conducting the study. The informed consent form was incorporated into the questionnaire, and the participants were required to confirm the consent form electronically. To maintain participant confidentiality, we did not collect names, emails or other contact details. In the final dataset, we assigned a unique random code for each valid response. The Ethics Committee of East West University has authorized the current study under the code (EWU/IQAC/DSR/ERC/2024/07‐15).

## Consent

All participants gave their consent to participate in the study.

## Conflicts of Interest

The authors declare no conflicts of interest.

## Transparency Statement

The lead author Md Sanaul Haque Mondal affirms that this manuscript is an honest, accurate, and transparent account of the study being reported; that no important aspects of the study have been omitted; and that any discrepancies from the study as planned (and, if relevant, registered) have been explained.

## Data Availability

The data that support the findings of this study are available from the corresponding author upon reasonable request.
